# Practical Points about Surgical Dressings

**Published:** 1890-04

**Authors:** 


### PRACTICAL POINTS ABOUT SURGICAL DRESSINGS.

A Vander Veer (*Albany Medical Annals*), in a report of four months' service in the Albany hospitals, says, in regard to dressings, that the methods were simple and the antiseptic agents neither new nor novel. The gauze was plain, of home manufacture, and medicated, as a rule, with bichloride of mercury. He bought his absorbent gauze in two hundred-yard lots, cut and folded this in five-yard pieces, and prepared for use. It was put in the following solution for twelve hours:

℞

| | |
|---|---|
| Bichloride of mercury - - - | 1 part. |
| Tartaric acid - - - - | 15 parts. |
| Glycerine - - - - - | 150 " |
| Water sufficient for - - - | 1000 " |

Eosin enough to give slight tint.—*M.*

After removal it was packed in stoneware jars, ready for use. The bihcloride gauze was used for making "Gamgee" pads for bandages, and for iodoform gauze by rubbing in its mesh. Iodoform and boric acid were used in dressing ulcers, both in powder and in ointment. Boric acid solutions were used in washing the bladder and urethra before and after operations. A onehalf per cent. solution of hydrogen proxide, he says, was very satisfactorily used about the mouth and nose. It acts also as a powerful deodorant.

In his abdominal work hot water took the place of all antiseptics, except in the dressing. The spray was used in the room for three days before opening the abdomen. No poisonous effects were observed during the four months from the use of antiseptics, except in one case, in which a slight idoform erythema appeared upon the abdomen after an abdominal section.—*Gaillard Medical Journal.*

